# X-Ray Structures, Intermolecular Interactions, and Structural Transformations of Dihydroquercetin Solvates and Polymorphs

**DOI:** 10.3390/pharmaceutics17121512

**Published:** 2025-11-23

**Authors:** Xin Meng, Yao Zou, Shiying Yang, Cheng Xing, Ningbo Gong, Guanhua Du, Yang Lu

**Affiliations:** 1 Beijing Key Laboratory of Innovative Drug Discovery and Polymorphic Research for Cerebrovascular Diseases, Chinese Academy of Medical Sciences and Peking Union Medical College, Beijing 100050, China; s2023008153@pumc.edu.cn (X.M.); 20180221156@bucm.edu.cn (Y.Z.); ysy@imm.ac.cn (S.Y.); xingc@imm.ac.cn (C.X.); 2 Beijing City Key Laboratory of Drug Target Identification and Drug Screening, Institute of Materia Medica, Chinese Academy of Medical Sciences and Peking Union Medical College, Beijing 100050, China; dugh@imm.ac.cn

**Keywords:** dihydroquercetin, solvates, polymorphs, crystal structure, physical characterization, hirshfeld surface, energy frameworks

## Abstract

**Background/Objectives**: Dihydroquercetin, known for its broad biological activities, is a key component in dietary supplements and functional foods. This study aims to identify its novel pure solid forms, advancing understanding of its physicochemical properties and polymorphism. **Methods**: Systematic screening, preparation, and characterization efforts identified five solvates: dihydroquercetin monohydrate (1:1, S1 and S2), sesquihydrate (1:1.5, S3), dihydrate (1:2, S4), and ACN solvate (1:1, S5), along with one solvent-free phase (S6). **Results**: The crystal structures of the five solvates were successfully elucidated for the first time. A comprehensive suite of techniques, including single-crystal and powder X-ray diffraction, DSC, TG, and FT-IR, were employed to characterize the solvates and polymorphs. Hirshfeld surface analysis, void map analysis, intermolecular energy calculations, and energy framework methods were utilized to investigate the characteristics of the solvates. The crystal transformation relationships among these forms were also explored. **Conclusions**: Results demonstrate that O···H interactions dominate the intermolecular forces, accounting for over 35% of the total interactions.

## 1. Introduction

In the fields of pharmaceutical science, materials chemistry, and nutraceuticals, the discovery of novel solid forms of active pharmaceutical ingredients (APIs) is of paramount importance [1]. These forms can enhance solubility, stability, and bioavailability, thereby improving drugability or the adaptability of natural products for medicinal applications [2,3]. Among strategies to overcome limitations of active pharmaceutical ingredients (APIs), polymorphic studies are the most direct and efficient. Compared to alternatives like chemical modification [4], they provide a simpler path to drug development while maintaining the inherent pharmacological activity. The investigation of solvates offers critical insights into intermolecular interactions and structure–activity relationships [5]. In certain instances, solvates represent the sole viable route to access specific polymorphic forms or hydrates [6]. By controlling crystallization parameters, researchers can selectively crystallize desired solvate structures [7]. These advancements optimize manufacturing processes and contribute to crystal engineering, which is foundational for designing novel materials and pharmaceuticals, thus driving innovations in drug development and nutraceutical applications [8,9].

**Figure 1:**
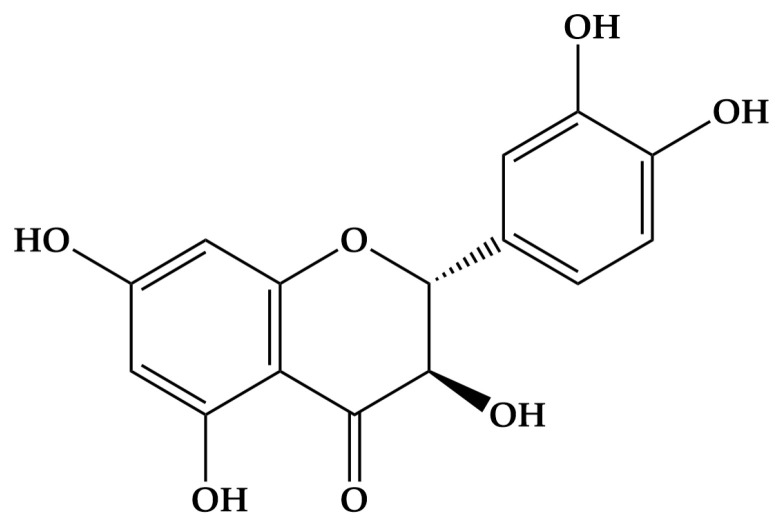
Chemical structures of (R,R)-enantiomer DHQ.

Dihydroquercetin (DHQ, C15H12O7, Figure 1), also known as taxifolin, is a natural flavonoid widely distributed in fruits, vegetables, and many plants [10]. As a key component of dietary supplements and functional foods, DHQ is highly valued for its rich antioxidant properties. In 2017, the European Commission issued (EU) 2017/2079, authorizing taxifolin-rich extract as a novel food ingredient and approving its use at a daily intake of 100 mg in dietary supplements, with the exception of infants, children, and adolescents [11]. Sunil summarized the health-promoting effects of taxifolin in 2019. Extensive studies have demonstrated that DHQ exhibits a wide range of biological activities, including antioxidant [12,13], anti-inflammatory [14,15], anti-anaphylaxis [16], antiradical [17], anti-Alzheimer [18], antimicrobial [19], antiangiogenic [20], and hepatoprotective properties [19]. Furthermore, it plays a crucial role in treating serious diseases including cancers [21], chronic obstructive pulmonary disease [22], pulmonary fibrosis [23], parkinsonism [24], obesity [25], periodontitis [26], cardiovascular [27], diabetic [28], and nervous disorders [29]. Additional research also indicates its therapeutic potential for diabetic encephalopathy [30], nephroprotective effects against acute kidney injury [31], and amelioration of ulcerative colitis [32]. These multiple biological effects and health benefits indicate that DHQ is highly suitable for use as a food supplement, functional food ingredient, and potential therapeutic agent.

DHQ exhibits polymorphism, with various crystalline forms reported. Selivanova first reported the Form I of DHQ in 1999 [33], a 2.5-hydrate crystallizing in the C 2 space group with the cell parameters a = 23.557 Å, b = 5.206 Å, c = 25.512 Å, β = 103.18 Å, and V = 3046.30 Å^3^. This structure was later confirmed by Nifant’ev and Terekhov in 2006 [34] and 2019 [35], respectively. In 2018, Wu [36] et al. reported two anhydrous forms of DHQ: Form II (melting point 216.8 °C) and Form III (melting point 239.2 °C). In 2020, Terekhov reported two crystalline forms: microspheres (amorphous, Form IV) and microtubes (pseudopolymorphism of Form I, designated as Form Ia) [37]. In 2021, Moura explored DHQ polymorphs and acquired six different crystalline phases of DHQ [38]. Although six DHQ phases have been described, most recrystallized samples were not phase-pure but were mixtures of two or more polymorphs or solvates. Only two phases have well-defined pure crystalline forms—Phase 1 (Form I) and Phase 3 (Form V)—determined by single-crystal X-ray diffraction (SCXRD).

Here, we present the screening, preparation, and characterization of DHQ solvates and polymorphs. Six pure phases were obtained, including four hydrates S1–S4 (Forms IX–XII), one solvate S5 (Forms VIII), and one anhydrate S6 (Forms V), with five crystal structures identified by SCXRD for the first time. All these crystalline phases are pure forms which are different from the reference [37]. All DHQ solid forms reported in the literature and newly discovered in this study are summarized in Table 1. The pure crystallized forms were characterized using PXRD, differential scanning calorimetry (DSC), thermogravimetric analysis (TGA) and Fourier transform infrared (FTIR). Transformation pathways among S1–S6 were identified. Hirshfeld surface analysis, packing efficiency analysis, intermolecular energy calculations, and 3D energy framework were utilized to investigate the five solvates’ properties, aiding in observing compound organization in crystal structures and quantifying intermolecular interaction contributions to stable crystal packing [39,40].

## 2. Materials and Methods

### 2.1. Materials

DHQ sesquihydrate (S3, purity: 98%, a racemic mixture of (R,R)- and (S,S)- enantiomers) was purchased from Shaanxi Huike Plant Development Co., Ltd., (Xi’an, China), ligustrazine hydrochloride (98%) was purchased from Wuhan Yuancheng science and technology development Co., Ltd., (Wuhan, China). All solvents (including methanol; ethanol; n-butanol; n-propanol; isopropanol; ethyl acetate; acetone; tetrahydrofuran; 1,4-dioxane; acetonitrile; chloroform; DMF; DMA; DMSO; purified water) used for experiments were of analytical or chromatographic grade purchased from sinopharm chemical reagent Co., Ltd. (Beijing, China) and Fuchen Chemical Reagent Co., Ltd. (Tianjin, China).

### 2.2. Phase Screening

The phase screening of DHQ was performed using the Technobis Crystal 16^®^ system. Based on the polarity of the solvents and the solubility of dihydroquercetin (DHQ), and considering the principles of appropriate boiling points and the ability to form supramolecular synthons, a systematic phase screening of DHQ was conducted using 13 solvents, notably including methanol, ethanol, acetone, acetonitrile, and ethyl acetate—at varying ratios and temperatures. About 50–200 mg of DHQ were added in 7 mL glass vials with different solvents; all solutions were prepared on a 5 mL scale. Solutions were agitated at 500 rpm using a magnetic stirrer bar at 55 °C during the experiments. The crystallization was induced by slow evaporation at room temperature. Specifically, the solutions were kept under ambient conditions allowing for gradual solvent evaporation. The resulting precipitates were isolated by filtration using filter paper and subsequently dried at room temperature in a well-ventilated area for 24 h. Each preparation method described above was performed three times to ensure the stability and reliability of the preparation process through quality control measures.

### 2.3. Preparation of Phases

The preparation of DHQ monohydrate powder S1 was performed by grinding 100 mg of DHQ raw material (S3, DHQ•1.5H_2_O) with 1 mL of acetone or methanol at 22 °C for 15 min. For single crystal S1, 50 mg of DHQ was dissolved in 5 mL acetone to form a supersaturated solution at 55 °C, filtered, and left at 20 °C for 10 days. S2 was prepared by dissolving 100 mg of DHQ raw material in acetonitrile or a 1:1 mixture of acetonitrile and acetone at 55 °C, followed by filtration and crystallization at room temperature for approximately 10 days. The formation of monohydrates S1 and S2 from ostensibly anhydrous organic solvents can be explained by the dehydration of the DHQ raw material (S3, DHQ•1.5H_2_O) during the crystallization process. DHQ sesquihydrate (S3) was identified from raw material. Single crystal S3 was prepared by dissolving 50 mg of DHQ in a 2:1 acetone–water mixture (about 4 mL) at 55 °C, followed by filtration crystallization at 20 °C for 10 days. For S4, about 50 mg of DHQ raw material was dissolved in ethanol containing ligustrazine hydrochloride, making a supersaturated solution at 45 °C, followed by filtration and storing at 25 °C for two weeks. For S5, 50 mg of DHQ raw material was weighed into a vial; a saturated acetonitrile solution (about 4 mL) was prepared at 55 °C, cooled to room temperature (about 15 °C), filtered, and 2 mL of the filtrate was mixed with 1 mL of chloroform. It was then left at room temperature for 3 days to obtain pale yellow flake crystals. DHQ anhydrate (S6) was prepared by drying DHQ raw material (S3, DHQ•1.5H_2_O) at 145 °C for 40 min. However, because S4 was serendipitously discovered during attempts to grow cocrystals of ligustrazine hydrochloride and DHQ, this phase could not be reproducibly obtained through subsequent preparation attempts. Therefore, only SCXRD analysis, Hirshfeld surface investigation, and related calculations were performed on the initially acquired sample.

### 2.4. SCXRD

The SCXRD measurements were conducted using a MicroMax 002+ diffractometer (Rigaku, The Woodlands, TX, USA) and an XtaLAB Synergy R DW system (The Woodlands, TX, USA) with Cu Kα radiation (λ = 1.54187 Å). The instruments were equipped with CCD detectors and a graphite monochromator (Rigaku, Americas, The Woodlands, TX, USA) to ensure high-precision data collection and optimal signal quality. Data collection and reduction were carried out using the CrysAlisPro program. The structure was solved using the direct method implemented in SHELXT and refined by SHELXL’s full matrix least-squares F2 procedure using SHELXL-2019/2 [41]. Non-hydrogen atoms were refined with anisotropic displacement parameters, while hydrogen atoms of the skeleton were refined with isotropic displacement parameters. Partial H atoms of the hydroxyl groups were located from the difference electron density maps, whereas other H atoms of water were theoretically assigned in calculated positions. Hydrogen atoms were placed in idealized positions with Uiso(H) = 1.2Ueq (CH) and Uiso(H) = 1.5Ueq(OH) [42]. Disordered moieties in the crystal structures were modeled using PART instructions. The geometries and displacement parameters were restrained using SIMU, RIGU, SADI, and DFIX commands to ensure a stable refinement. Crystallographic data for DHQ•monohydrates, DHQ•sesquihydrate, DHQ•dihydrate, and DHQ•acetonitrile solvate have been deposited at the Cambridge Crystallographic Data Centre as No. 2422558–2422562, respectively.

### 2.5. Hirshfeld Surface Analysis and Packing Efficiency Analysis

Hirshfeld surfaces and fingerprint plots were obtained using CrystalExplorer 21.5 [43,44]. Void Map Calculations were calculated using Mercury software (version 2020.3.0, Cambridge Crystallographic Data Center, Cambridge, UK) [45]. The calculation parameters were set to a probe radius of 1.0 Å and an approximate grid spacing of 0.3 Å, with the calculations performed using the contact surface method.

### 2.6. Intermolecular Energy Calculation and Their Energy Frameworks

The intermolecular energy was estimated and visualized [46] with the CrystalExplorer 25.09 software; the single-point molecular wave functions were calculated using the B3LYP/def2-svp [47] method with a cluster radius of 20 Å. Energies of intermolecular model were also analyzed using the energy framework method implemented in CrystalExplorer 21.5 [39,43,44,46]. A pair of molecules was selected to calculate the Pairwise Energy of DHQ solvates. The total interaction energy (Etot) is typically calculated by Equation (1)

Etot = keleEele + kpolEpol + kdisEdis + krepErep
(1)

where the components of Eele, Epol, Edis, and Erep represented the energies of electrostatic interactions, polarization, dispersion and exchange–repulsion, with corresponding scale factors *k* of 1.057, 0.740, 0.871, and 0.618, respectively [47].

### 2.7. PXRD

Powder X-ray diffraction data were collected by a D/max-2550 diffractometer (Rigaku, Tokyo, Japan) with Cu Ka radiation (λ = 1.54178 Å), operated at 40 kV and 150 mA. Samples were determined at the diffraction angle (2θ) from 3 to 40° with a scanning rate of 8°/min and a step size of 0.02°.

### 2.8. DSC and TGA

DSC was performed in a DSC 1 system (Mettler-Toledo, Greifensee, Switzerland) to determine the melting temperature and the thermodynamic properties of the samples. Samples (3–5 mg) were placed in sealed aluminum pans with a pinhole and heated at a heating rate of 10 °C/min under nitrogen flow (50 mL/min). An empty pinhole disk was used as a reference. TGA was performed in a TGA/DSC system (Mettler Toledo, Switzerland). Samples (5–10 mg) were placed in standard alumina crucibles and heated from 30 to 500 °C at a rate of 10 °C/min under the protection of a nitrogen flow of 10 mL/min.

### 2.9. FT-IR

FT-IR spectra were obtained using an FT-IR spectrophotometer (Spectrum 400, PerkinElmer, Waltham, MA, USA). Powders were gently ground with a mortar and put on a diamond plate of the ATR accessory devices. All samples were scanned in the range of 4000–650 cm^−1^ at a resolution of 4 cm^−1^.

### 2.10. Accelerated Stability Study

The solid-state stability of the crystalline phases was investigated under controlled stress conditions. Three separate stability studies were conducted in a pharmaceutical stability testing chamber (HWS-70B, Taisite Instrument Co., Ltd., Tianjin, China) with precisely defined parameters: thermal stress study (60 °C ± 2 °C, 60% ± 5%, no light); humidity stress study (25 °C ± 2 °C, 90% ± 5%, no light); and photolytic stress study (25 °C ± 2 °C, 60% ± 5%, 4500 ± 500 lx). All samples were placed in transparent glass bottles. The study period was 10 days under all conditions, with sampling and PXRD analysis conducted on days 0, 5, and 10 to monitor solid-state phase transitions.

## 3. Results and Discussion

### 3.1. Crystal Structures

**Figure 2:**
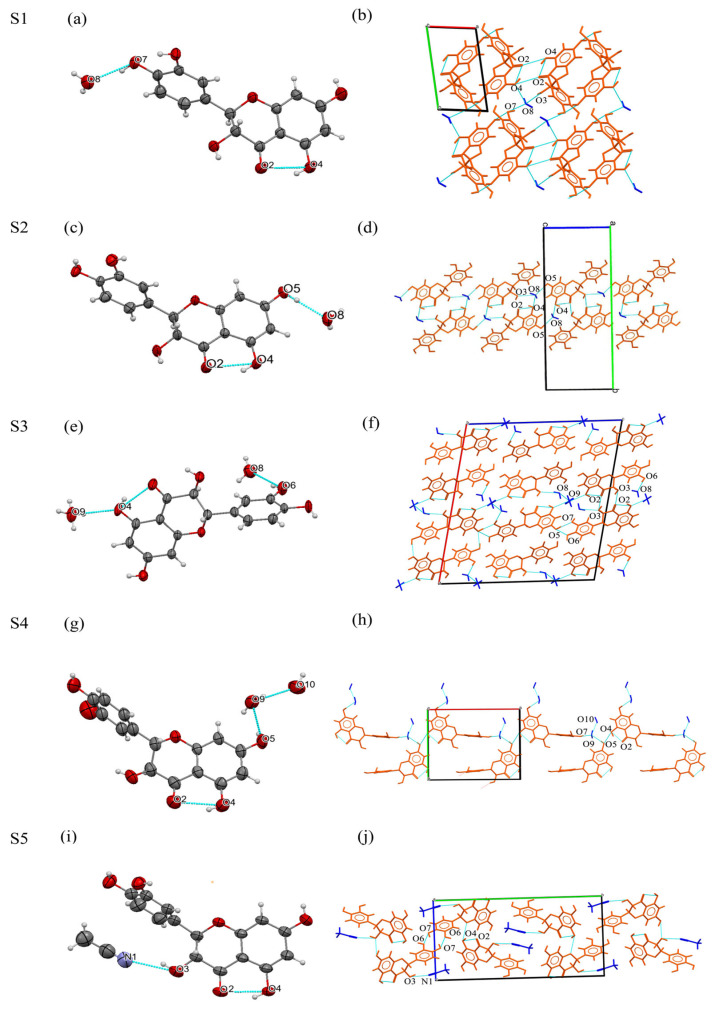
The crystal structure and hydrogen bond interactions of DHQ solvates (**b**,**d**,**f**,**h**,**j**). The orange structures represent DHQ molecules, while blue structures represent solvent molecules (water or acetonitrile). The boxes formed by the axes represent the unit cells. The letters label specific atoms involved in key hydrogen bonding interactions. The ellipsoids are drawn at probability levels of 50% for (**a**,**c**,**e**,**g**,**i**).

S1 DHQ monohydrate. The crystal structure of DHQ monohydrate S1 consists of a water molecule and a DHQ molecule in a 1:1 ratio (Z′ = 1, Figure 2a). Based on chromone skeleton, two DHQ molecules plus two water molecules form an $R_{4}^{4}(18)$ motif, while four molecules (two DHQ and two water) create an $R_{4}^{2}(22)$ motif [48]. These units collectively construct a net structure extending infinitely along the a-axis (Figure 2b). Notably, among all hydrogen bonds in the structure, the hydrogen bond O7–H7···O8, which is formed between different types of molecules, DHQ and a water molecule, exhibits the shortest H···A distance (2.66Å) and the largest donor acceptor angle (162.44°), indicating that it is stronger and more linear compared to other hydrogen bonds, which confirms that DHQ readily forms solvates and that the presence of water molecules plays a crucial role in stabilizing the crystal structure of S1 [3]. The geometrical parameters of the hydrogen bonds in the crystal structures are detailed in Table S1.

S2 DHQ monohydrate. The crystal structure of DHQ monohydrate S2 consists of a water molecule and a DHQ molecule in a 1:1 ratio (Z′ = 1, Figure 2c). The DHQ molecule exhibits disorder over two positions with an occupancy of 83%:17%. Based on benzene ring C skeleton, two DHQ molecules and two water molecules form an $R_{4}^{4}(16)$ motif, and based on chromone skeleton and benzene ring C skeleton, two DHQ molecules plus two water molecules form an $R_{4}^{4}(18)$ motif [49]. And the hydrogen bond O5–H5···O8 formed between DHQ and a water molecule, exhibits the shortest H···A distance (2.70Å) and the largest donor acceptor angle (172.37°). These motifs collectively construct a net structure that extends infinitely along the b-axis (Figure 2d).

S3 DHQ•1.5H_2_O. The crystal structure of DHQ•1.5H_2_O S3 comprises DHQ and water in a 1:1.5 ratio. (Z′ = 1, Figure 2e). The DHQ molecule exhibits disorder over two positions with an occupancy of 86%:14%. The asymmetric cell comprises four independent molecules, interconnected through OH···O–C hydrogen bonds. A dimeric arrangement of two DHQ molecules forms an $R_{2}^{2}(10)$ motif linked via an O3–H3A···O2 (2.772 Å) hydrogen bond [50]. Additionally, two adjacent DHQ molecules form a larger cycle through an O7–H7···O5 (2.809 Å) hydrogen bond. And the hydrogen bond O6–H7···O8 formed between DHQ and a water molecule exhibits the shortest H···A distance (2.69 Å) and the largest donor acceptor angle (166.07°). These structural units collectively form a net structure that extends infinitely along the c-axis (Figure 2f).

S4 DHQ•2H_2_O. The crystal structure of DHQ•2H_2_O S4 comprises DHQ and water in a 1:2 ratio. (Z′ = 1, Figure 2g). The DHQ molecule is disordered over two positions with an occupancy of 59%:41%. Based on chromone skeleton and benzene ring C skeleton, the structural motifs include two DHQ molecules and two water molecules form an $R_{2}^{2}(16)$ motif, two DHQ molecules with four water molecules form an $R_{4}^{4}(22)$ motif, and three DHQ molecules and three water molecules form an $R_{4}^{4}(18)$ motif and an $R_{4}^{4}(16)$ motif [51]. And the hydrogen bond O5–H5···O9 formed between DHQ and a water molecule, exhibits the shortest H···A distance (2.76 Å) and the largest donor acceptor angle (176.07°). These units construct a net structure that extends infinitely along the a-axis (Figure 2h).

S5 DHQ•ACN. The crystal structure of DHQ•ACN S5 comprises DHQ and acetonitrile in a 1:1 ratio (Z′ = 1, Figure 2i). The DHQ molecule is disordered over two positions with an occupancy of 70%: 30%, and the acetonitrile molecule is disordered with a 92%:8% occupancy. Two DHQ molecules form into dimer and an $R_{2}^{2}$(10) motif linked by O7–H7···O6 (2.703 Å) [50], An acetonitrile molecule interacts with DHQ molecule by hydrogen bond O3–H3A···N1 (2.767 Å). Additionally, two adjacent DHQ molecules linked each other by O6–H6···O4 (2.762 Å). These units collectively construct a net structure extending infinitely along the b-axis (Figure 2j).

Crystal data and structure refinement parameters for these five DHQ solvates are summarized in Table 2.

### 3.2. Hirshfeld Surface Investigation and Packing Efficiency Analysis

**Figure 3:**
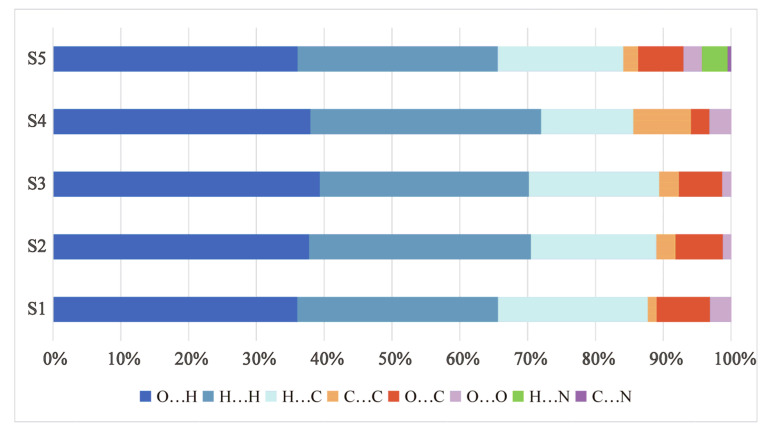
The contribution of different intermolecular interactions to the Hirshfeld surface of the DHQ solvates.

**Figure 4:**
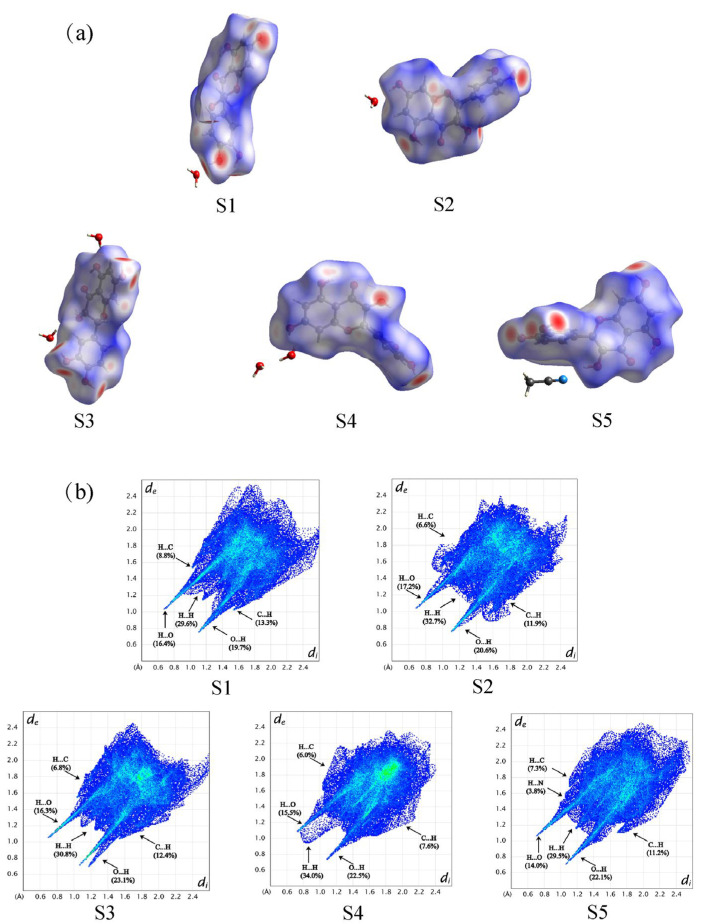
(**a**) The Hirshfeld surface and (**b**) 2D fingerprint plots of DHQ solvates.

**Figure 5:**
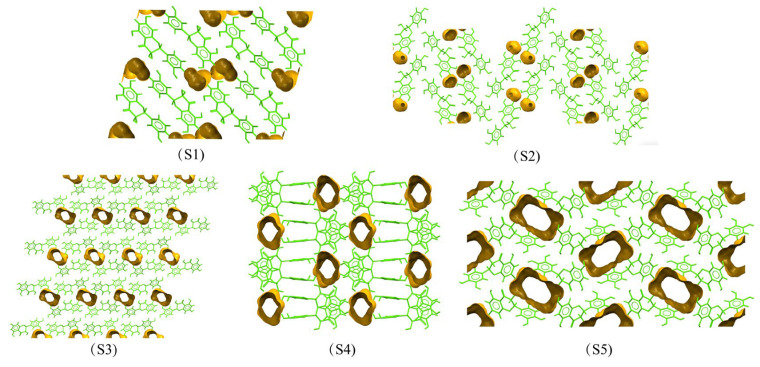
Void maps in the crystal structures of DHQ solvates.

Figure 3 illustrated the contributions of various interactions to the Hirshfeld surface of DHQ solvates. Among these, the O···H interactions were the most significant intermolecular interaction, followed by H···H and C···H interactions. Figure 4 presents the Hirshfeld surface and 2D fingerprint plots of DHQ solvates. The deep red spots indicate the locations where hydrogen bonds form [52], with the strength of a hydrogen bond being inversely proportional to the distance between the donor and acceptor, resulting in deeper red spots and larger diameters on the Hirshfeld surface [53]. In S1–S4, the hydroxyl group of DHQ forms hydrogen bonds with water molecules, whereas in S5, the hydroxyl group of DHQ forms hydrogen bonds with the cyano nitrogen atom. In the 2D fingerprint plots, the O···H interactions are depicted as prominent spikes in the lower left region, H···H interactions appear as dispersed points in the middle, and C···H interactions exhibit a wing-like pattern. These interactions are critical to the stability of DHQ solvates. Moreover, solvent molecules in a solvate enhance the packing efficiency [54,55]. To visualize and compare the voids within the crystal structures, Mercury software was utilized. The void map results shown in Figure 5 reveal that void channels are absent in the crystal structures of S1 and S2, where solvent occupies only 7.3% (49.68 Å^3^) and 4.1% (56.73 Å^3^) of the unit cell volume, respectively. In contrast, void open channels are present in S3, S4, and S5, occupying 10.8% (308.77 Å^3^), 13.1% (194.19 Å^3^), and 20.7% (324.29 Å^3^) of the unit cell volume, respectively. In general, the desolvation process is intricately linked to the type of voids, their proportion within the material, and the robustness of the hydrogen bond network [56].

### 3.3. Intermolecular Energy Calculation and Energy Framework of DHQ Solvates

**Figure 6:**
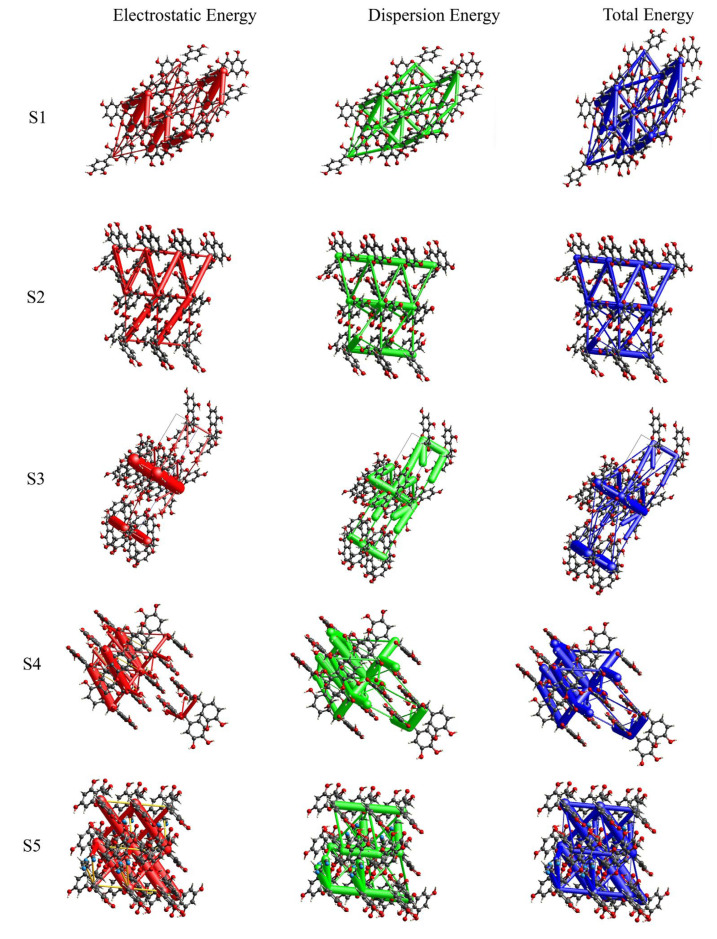
Energy frameworks of DHQ solvates.

The intermolecular energy calculation results are presented in Table 3, with detailed interaction energies provided in Tables S2–S6. The data reveal that in structures S1, S2, and S3, electrostatic interactions are the predominant forces, outweighing polarization, dispersion, and exchange–repulsive interactions. It is demonstrated that hydrogen bonding interactions serve as the primary force stabilizing their structure. In contrast, for structures S4 and S5, dispersion interactions dominate over other energy contributions, which is characteristic of many small molecules with a limited number of strong hydrogen-bond donors and acceptors [57]. Furthermore, the non-directionality of dispersion forces further complicates the prediction of crystal structures [58]. The energy framework allows for an intuitive understanding of the contributions of energy to crystal stability. Figure 6 illustrates the energy framework of DHQ, with different colors representing various types of energy: red for Eele, green for Edis, and blue for Etot of the molecule. Intermolecular interaction energies are depicted using cylinders, where the radius of each cylinder is proportional to the magnitude of the interaction energy in that direction. In the visual representation of S1–S5, the green cylinders, which symbolize dispersive forces, are noticeably larger than the red cylinders representing electrostatic forces. This visual disparity confirms the significant contribution of dispersive forces to the stability of the molecular structure. And the relative sizes of the cylinders effectively illustrate the calculated energy differences among the DHQ solvates. Additionally, in all energy frameworks, S3 stands out as it contains the highest number of energy data points in Table S4. This increased energy contribution is instrumental in enhancing the stability of S3 [50,59]. Compared to the DSC desolvation enthalpy, a positive correlation exists between the energy and the enthalpy for the monohydrates S1 and S2, which share an isostructural framework. In contrast, the pronounced channel-like voids in S3, S4, and S5, which constitute a significant portion of the unit cell volume, allow solvent molecules to escape more readily, resulting in their lower desolvation enthalpies.

### 3.4. PXRD Analysis

**Figure 7:**
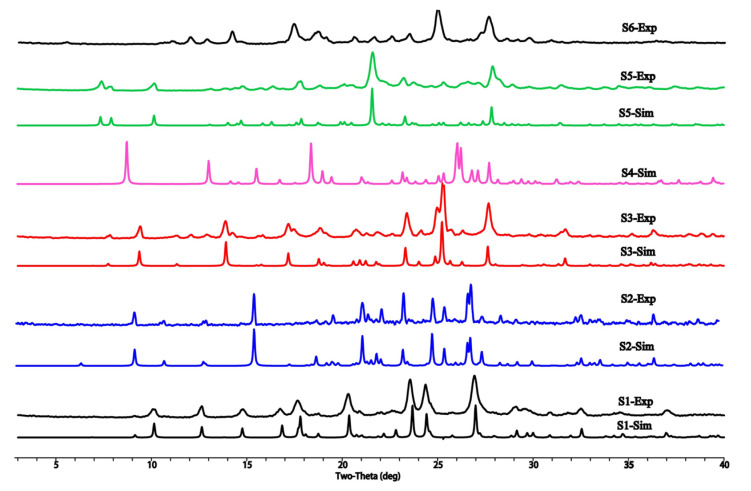
Powder X-ray diffraction patterns of DHQ solvates and polymorphs.

Different crystalline phases of DHQ, including S1–S6, were characterized through powder X-ray diffraction. The experimental and the simulated PXRD patterns of the crystals based on the crystallographic data were also shown in Figure 7. It is evident that S1–S6 exhibited distinct peaks, indicating they were solvates and polymorphs. Moreover, the remarkable correspondence between the simulated PXRD patterns and the experimental measurements underscores the high purity of these phases.

### 3.5. DSC Analysis

**Figure 8:**
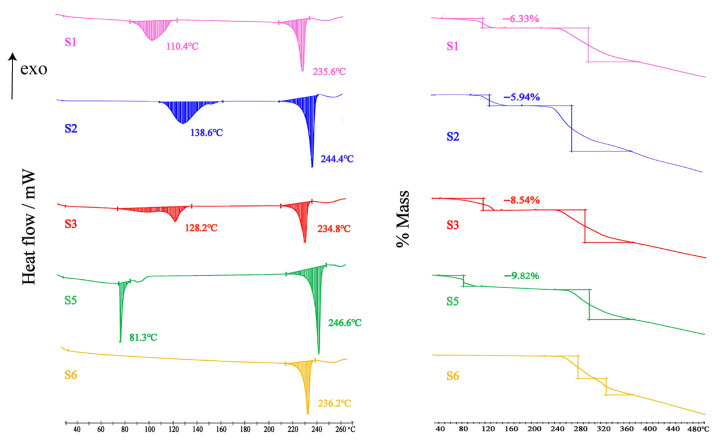
DSC and TG curves of DHQ solvates and polymorphs.

Differential scanning calorimetry (DSC) is a thermal analysis technique widely used to determine the melting points of compounds, distinguishing between polymorphs and solvates, and studying the phase transitions of substances [60]. The DSC profiles of those DHQ solvates and polymorphs are presented in Figure 8. The DSC curves of S1, S2, S4, and S5 exhibited similar features with an initial endothermic peak (110.4 °C, S1; 138.6 °C, S2; 128.2 °C, S3; 81.3 °C, S5), followed by a second endothermic peak (235.5 °C, S1; 244.4 °C, S2; 234.8 °C, S3; 246.6 °C, S5), which implied that there was a desolvent endothermic peak before the melting endothermic peak. This inference was further supported by the TG thermograms. And S6 showed one strong melting endothermic peak at 236.2 °C (Tonset = 232.5 °C; ΔH = −42.6 KJ/mol).

### 3.6. TGA Analysis

As shown in Figure 8, the stoichiometry of compound solvent was also obtained by mass loss at programmed heating with a thermal gravimetric analyzer. The TG curves S1, S2, S3, and S5 showed both the exothermic step of solvent and melting decomposition steps of DHQ. Within the corresponding temperature ranges, the mass loss rates during the initial weight loss stages for S1, S2, S3, and S5 were 6.33%, 5.94%, 8.54%, and 9.82%, respectively. As shown in Table 4, the experimental mass loss rates matched the theoretical desolvation mass fractions, confirming the stoichiometric ratios of solvent to DHQ in the single-crystal structures as 1:1, 1:1, 1:1.5, and 1:2, respectively. In contrast, S6 displayed only the melting decomposition steps of DHQ, with no characteristic weightless step of solvates observed.

### 3.7. IR Analysis

**Figure 9:**
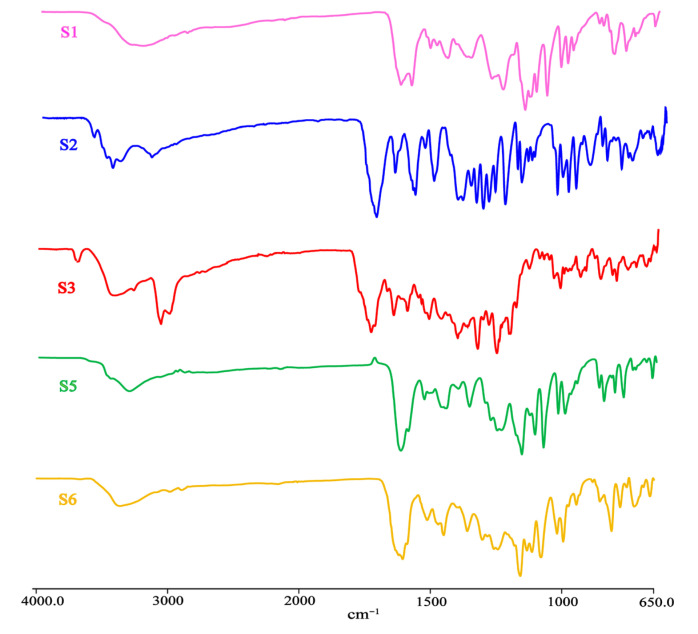
FT-IR spectra of DHQ solvates and polymorphs.

We performed Fourier-transform infrared (FT-IR) spectroscopic analysis to characterize and elucidate the noncovalent interactions present in the DHQ solvates and polymorphs. Generally, flavonoids exhibit characteristic O–H stretching absorption, which serves as a critical region for distinguishing different solid forms. As shown in Figure 9, all samples display significant absorption peaks in this region. Compared to S6, the other solvates show varying degrees of red-shift and band broadening in their O–H stretching vibrations due to the introduction of solvent molecules and the resulting increase in hydrogen bonding. Notably, S3, which contains the highest number of water molecules and forms a stronger or more extensive hydrogen-bonding network, exhibits a markedly red-shifted O–H stretching peak with significantly greater intensity than the other samples.

The strongest absorption peak, observed at ~1620 cm^−1^, is a signature band of flavonoids and is attributed to the C=O stretching vibration [61,62,63]. The adjacent peaks at 1550 cm^−1^ and 1450 cm^−1^ correspond to aromatic ring (C=C) skeletal vibrations, confirming the presence of the phenyl structure. In the fingerprint region (1500–650 cm^−1^), complex vibrations including phenolic C–O stretching and C–O–H bending modes are observed.

### 3.8. Phase Transformations

**Figure 10:**
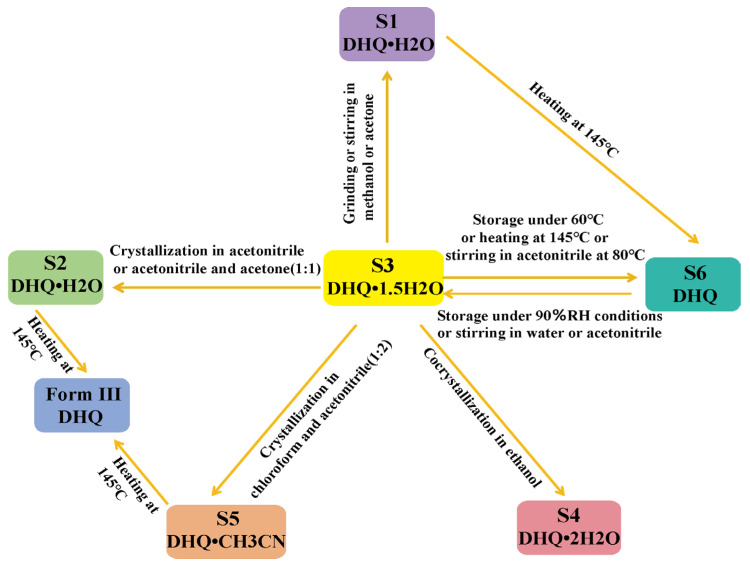
The transformation of DHQ solvates and polymorphs.

The phase transformation behavior was investigated and analyzed using PXRD. The transformation relationships are depicted in Figure 10 and can be summarized as follows. (i) S3 can be converted to S1 through grinding or stirring in methanol or acetone as the solvent medium; (ii) S3 undergoes recrystallization in different solvent media, resulting in S1, S2, and S5; (iii) S3 is prepared as S4 via cocrystallization in ethanol; (iv) S3, when stored at 60 °C, heated to 145 °C, or stirred in acetonitrile at 80 °C, changes into S6. Conversely, S6 reverts to S3 when exposed to 90% relative humidity (RH) or when stirred in water or acetonitrile; (v) S1 is transformed into S6 by heating to 145 °C. S2 and S5 are altered to Form III under the same heating conditions. Previous experimental results also indicate that the hydrated phase of DHQ can convert to the anhydrous phase. However, due to the differing crystallization routes and solvents employed, we did not obtain Form I (DHQ 2.5 hydrate) during the experiments. These results highlight the phase transformation behavior of DHQ, which is influenced by various experimental conditions, making its crystal structures challenging to predict. And it is consistent with energy framework calculations, where dispersion energy dominates over electrostatic energy [38,58].

## 4. Conclusions

In conclusion, a series of pure phases with DHQ including four hydrates, one solvate, and one anhydrate were obtained, which were structurally identified and characterized in this work. The five DHQ solvates could be determined, allowing exploration of their unit cell parameters, Hirshfeld surface analysis, void map analysis, intermolecular energies, and crystal transformation relationships. SCXRD analysis reveals that the hydrogen bonds formed between DHQ and water molecules in S1–S4 are stronger and more linear than other hydrogen bonds, confirming the crucial role of water molecules in stabilizing the crystal structure of DHQ. Hirshfeld surface analysis confirmed that the O···H interaction was the primary intermolecular force, while in S5, DHQ interacts with the solvent through N···H interactions, emphasizing the critical role of hydrogen bonds in DHQ solvates. Interaction energy calculations further revealed that electrostatic, polarization, repulsion, and dispersion energy components collectively contribute to the stability of DHQ solvates. Specifically, repulsion energies dominate in S1 and S3, while dispersion energies play a primary role in S2, S4, and S5.

The non-directional nature of these interactions may account for the propensity of DHQ to form both polymorphs and solvates. Additionally, this study investigated the transformation relationships among DHQ solid forms, providing critical insights to prevent unexpected phase transitions during storage. Despite these findings, this study has several limitations. First, while we identified the predominant intermolecular interactions, the precise kinetic factors governing polymorphic transformation pathways remain to be fully elucidated. Second, the energy calculations, while informative for understanding relative stability, cannot fully predict crystallization outcomes under various processing conditions. Finally, the potential impact of these solid forms on the biopharmaceutical properties of DHQ, such as dissolution behavior and oral bioavailability, warrants further investigation to establish clinically relevant correlations between solid-state characteristics and pharmaceutical performance.

## Figures and Tables

**Table 1:** Summary of Solid-State Forms of DHQ.

Form Designation	Category	Chemical Composition	Crystal System/State
Form I	Reported	DHQ•2.5H_2_O	Monoclinic
Form Ia	Reported	DHQ•xH_2_O (pseudopolymorph, microtubes)	Monoclinic
Form II	Reported	DHQ	Crystalline
Form III	Reported	DHQ	Crystalline
Form IV	Reported	DHQ (microspheres)	Amorphous
Form V	Reported	DHQ (S6)	Monoclinic
Form VI	Reported	DHQ•1H_2_O	Crystalline
Form VII	Reported	DHQ	Triclinic
Form VIII	Newly Discovered	DHQ•1ACN (S5)	Monoclinic
Form IX	Newly Discovered	DHQ•1H_2_O (S1)	Triclinic
Form X	Newly Discovered	DHQ•1H_2_O (S2)	Monoclinic
Form XI	Newly Discovered	DHQ•1.5H_2_O (S3)	Monoclinic
Form XII	Newly Discovered	DHQ•2H_2_O (S4)	Monoclinic

**Table 2:** Crystal data and structure refinement parameters for DHQ solvates.

Parameters	S1	S2	S3	S4	S5
Formula	C_15_H_12_O_7_· H_2_O	C_15_H_12_O_7_· H_2_O	C_15_H_12_O_7_· 1.5(H_2_O)	C_15_H_12_O_7_· 2H_2_O	C_15_H_12_O_7_· C_2_H_3_N
Formula weight	322.26	322.26	331.27	340.28	345.30
Temperature/K	293(2)	293(2)	293(2)	293(2)	293(2)
Crystal size (mm)	0.05 × 0.12 × 0.22	0.11 × 0.14 × 0.27	0.04 × 0.09 × 0.41	0.21 × 0.23 × 0.25	0.05 × 0.12 × 0.22
Description	plate	needle	plate	needle	plate
Crystal system	triclinic	monoclinic	monoclinic	monoclinic	monoclinic
Space group	P − 1	P 2_1_/n	C 2/c	P 2_1_/c	P 2_1_/c
a (Å)	5.378(1)	4.835(1)	25.927(1)	15.913(1)	5.15(1)
b (Å)	10.115(1)	27.649(1)	4.808(1)	13.145(1)	24.10 (1)
c (Å)	13.260(1)	10.293(1)	23.251(1)	7.119(1)	12.63(1)
α (°)	75.97(1)	90	90	90	90
β (°)	82.90(1)	95.51(1)	100.89(1)	93.71(1)	90.90(1)
γ (°)	79.06(1)	90	90	90	90
Volume (Å3)	684.83(4)	1369.62(6)	2846.41(9)	1486.01(5)	1566.81(4)
Z/Z′	2/1	4/1	8/1	4/1	4/1
Density (g/cm^3^)	1.563	1.563	1.546	1.521	1.464
Independent reflections	2601	2902	2779	2999	2989
Reflections with I > 2σ(I)	2512	2587	2301	2738	2506
Final R, wR(F^2^) value	0.050, 0.140	0.065, 0.177	0.083, 0.206	0.061, 0.148	0.082, 0.216
GOOF	1.070	1.059	1.042	1.039	1.091
Rint	0.0180	0.0907	0.0417	0.0203	0.0345
CCDC number	2,422,558	2,422,559	2,422,560	2,422,561	2,422,562

**Table 3:** Interaction energies (kJ mol^−1^) for selected contacts of DHQ solvates.

Variable	E_ele_	E_pol_	E_dis_	E_rep_	E_tot_
S1	−214.3	−32.0	−213.6	179.0	−336.2
S2	−247.6	−31.0	−212.9	164.8	−377.5
S3	−328.5	−36.2	−223.3	250.1	−400.0
S4	−158.1	−32.7	−212.9	153.3	−299.6
S5	−113.6	−36.3	−201.7	151.2	−364.2

**Table 4:** TGA Data for DHQ solvates.

Solvate	Desolvation Temperature (°C)	Theoretical Weight Loss (%)	Experimental Weight Loss (%)
S1	60–140	5.56	6.33
S2	110–160	5.56	5.95
S3	50–150	8.11	8.54
S5	60–150	11.81	9.82

## Data Availability

The data supporting the findings of this study are available from the corresponding authors upon reasonable request.

## Supplementary Materials

The following supporting information can be downloaded at https://www.mdpi.com/article/10.3390/pharmaceutics17121512/s1, Table S1: Hydrogen bond geometrical parameters of crystal structures; Table S2: Molecular Pairwise Interaction Energies (kJ/mol) Derived from the Energy Framework Calculation for S1 of DHQ; Table S3: Molecular Pairwise Interaction Energies (kJ/mol) Derived from the Energy Framework Calculation for S2 of DHQ; Table S4: Molecular Pairwise Interaction Energies (kJ/mol) Derived from the Energy Framework Calculation for S3 of DHQ; Table S5: Molecular Pairwise Interaction Energies (kJ/mol) Derived from the Energy Framework Calculation for S4 of DHQ; Table S6: Molecular Pairwise Interaction Energies (kJ/mol) Derived from the Energy Framework Calculation for S5 of DHQ. Table S7: FT−IR spectral frequencies of DHQ solvates and polymorphs.

## References

[1] Zhang B., Yang D., Zhang L., Hu K., Yang S., Lu Y., Du G. (2024). Experimental and Theoretical Investigation of Five Mosapride Forms. J. Mol. Struct..

[2] Fael H., Barbas R., Prohens R., Ràfols C., Fuguet E. (2021). Synthesis and Characterization of a New Norfloxacin/Resorcinol Cocrystal with Enhanced Solubility and Dissolution Profile. Pharmaceutics.

[3] Guo M., Sun X., Chen J., Cai T. (2021). Pharmaceutical cocrystals: A review of preparations, physicochemical properties and applications. Acta Pharm. Sin. B.

[4] Tretyakova I.S., Rychkov D.A., Kil’MEt’EV A.S., Lomovskiy I.O. (2023). Computational study of chemical phenol glycosylation mechanism in the gas phase for modeling direct glycoconjugate formation in raw plant material. Comput. Theor. Chem..

[5] Fu J., Zheng X., Lu X. (2014). Crystallization of Asiaticoside from Total Triterpenoid Saponins of Centella Asiatica in a Methanol + Water System. Ind. Eng. Chem. Res..

[6] Clements M., Blackie M., de Kock C., Lawrence N., Smith P., le Roex T. (2019). Investigation into the Structures and Properties of Multicomponent Crystals Formed from a Series of 7-Chloroquinolines and Aromatic Acids. Cryst. Growth Des..

[7] Yu H., Zhang B., Liu M., Xing C., He G., Zhang L., Gong N., Lu Y., Du G. (2023). Theoretical and experimental cocrystal screening of temozolomide with a series of phenolic acids, promising cocrystal coformers. Chin. Chem. Lett..

[8] Bhattacharya S., Sameena J., Saha B.K. (2011). Solvates of Ajmaline and Two-Dimensional Isostructurality between Methanol and Ethanol Solvates. Cryst. Growth Des..

[9] Islam S., Dey P., Seth S.K. (2024). Structural elucidation and various computational studies for quantitative investigation of intermolecular interactions in pyridine-2,6-dicarboxylic acid and its di-hydrate. J. Mol. Struct..

[10] Sunil C., Xu B. (2019). An insight into the health-promoting effects of taxifolin (dihydroquercetin). Phytochemistry.

[11] Products N.A.A. (2017). (.E.P.O.D.; Turck, D.; Bresson, J.; Burlingame, B.; Dean, T.; Fairweather-Tait, S.; Heinonen, M.; Hirsch-Ernst, K.I.; Mangelsdorf, I.; McArdle, H.J.; et al. Statement on the safety of taxifolin-rich extract from Dahurian Larch (*Larix gmelinii*). EFSA J..

[12] Kolhir V.K., Bykov V.A., Baginskaja A.I., Sokolov S.Y., Glazova N.G., Leskova T.E., Sakovich G.S., Tjukavkina N.A., Kolesnik Y.A., Rulenko I.A. (1996). Antioxidant Activity of a Dihydroquercetin Isolated from *Larix gmelinii* (Rupr.) Rupr. Wood. Phytother. Res..

[13] Zeng G., Wu Z., Cao W., Wang Y., Deng X., Zhou Y. (2018). Identification of anti-nociceptive constituents from the pollen of Typha angustifolia L. using effect-directed fractionation. Nat. Prod. Res..

[14] Wang Y.-H., Wang W.-Y., Chang C.-C., Liou K.-T., Sung Y.-J., Liao J.-F., Chen C.-F., Chang S., Hou Y.-C., Chou Y.-C. (2006). Taxifolin ameliorates cerebral ischemia-reperfusion injury in rats throughits anti-oxidative effect and modulation of NF-kappa B activation. J. Biomed. Sci..

[15] Luo P., Feng X., Liu S., Jiang Y. (2024). Traditional Uses, Phytochemistry, Pharmacology and Toxicology of *Ruta graveolens* L.: A Critical Review and Future Perspectives. Drug Des. Dev. Ther..

[16] Akinmoladun A.C., Oladejo C.O., Josiah S.S., Dele Famusiwa C., Ojo O.B., Olaleye M.T. (2018). Catechin, quercetin and taxifolin improve redox and biochemical imbalances in rotenone-induced hepatocellular dysfunction: Relevance for therapy in pesticide-induced liver toxicity?. Pathophysiology.

[17] Topal F., Nar M., Gocer H., Kalin P., Kocyigit U.M., Gülçin I., Alwasel S.H. (2016). Antioxidant activity of taxifolin: An activity–structure relationship. J. Enzym. Inhib. Med. Chem..

[18] Gunesch S., Hoffmann M., Kiermeier C., Fischer W., Pinto A.F., Maurice T., Maher P., Decker M. (2020). 7-O-Esters of taxifolin with pronounced and overadditive effects in neuroprotection, anti-neuroinflammation, and amelioration of short-term memory impairment in vivo. Redox Biol..

[19] Aires A., Marrinhas E., Carvalho R., Dias C., Saavedra M.J. (2016). Phytochemical Composition and Antibacterial Activity of Hydroalcoholic Extracts of *Pterospartum tridentatum* and *Mentha pulegium* against Staphylococcus aureus Isolates. BioMed Res. Int..

[20] Chen J., Sun X., Xia T., Mao Q., Zhong L. (2018). Pretreatment with dihydroquercetin, a dietary flavonoid, protected against concanavalin A-induced immunological hepatic injury in mice and TNF-α/ActD-induced apoptosis in HepG2 cells. Food Funct..

[21] Lin X., Dong Y., Gu Y., Kapoor A., Peng J., Su Y., Wei F., Wang Y., Yang C., Gill A. (2023). Taxifolin Inhibits Breast Cancer Growth by Facilitating CD8+ T Cell Infiltration and Inducing a Novel Set of Genes including Potential Tumor Suppressor Genes in 1q21.3. Cancers.

[22] Unver E., Tosun M., Olmez H., Kuzucu M., Cimen F.K., Suleyman Z. (2019). The Effect of Taxifolin on Cisplatin-Induced Pulmonary Damage in Rats: A Biochemical and Histopathological Evaluation. Mediat. Inflamm..

[23] Yuan L., Sun Y., Zhou N., Wu W., Zheng W., Wang Y. (2022). Dihydroquercetin Attenuates Silica-Induced Pulmonary Fibrosis by Inhibiting Ferroptosis Signaling Pathway. Front. Pharmacol..

[24] Akinmoladun A.C., Famusiwa C.D., Josiah S.S., Lawal A.O., Olaleye M.T., Akindahunsi A.A. (2022). Dihydroquercetin improves rotenone-induced Parkinsonism by regulating NF-κB-mediated inflammation pathway in rats. J. Biochem. Mol. Toxicol..

[25] Su H., Wang W., Zheng G., Yin Z., Li J., Chen L., Zhang Q. (2022). The anti-obesity and gut microbiota modulating effects of taxifolin in C57BL/6J mice fed with a high-fat diet. J. Sci. Food Agric..

[26] Alpan A.L., Kızıldağ A., Özdede M., Karakan N.C., Özmen Ö. (2020). The effects of taxifolin on alveolar bone in experimental periodontitis in rats. Arch. Oral Biol..

[27] Itaya S., Igarashi K. (1992). Effects of Taxifolin on the Serum Cholesterol Level in Rats. Biosci. Biotechnol. Biochem..

[28] Gao L., Yuan P., Zhang Q., Fu Y., Hou Y., Wei Y., Zheng X., Feng W. (2020). Taxifolin improves disorders of glucose metabolism and water-salt metabolism in kidney via PI3K/AKT signaling pathway in metabolic syndrome rats. Life Sci..

[29] Alam Q., Krishnamurthy S. (2022). Dihydroquercetin ameliorates LPS-induced neuroinflammation and memory deficit. Curr. Res. Pharmacol. Drug Discov..

[30] Cheng X., Huang J., Li H., Zhao D., Liu Z., Zhu L., Zhang Z., Peng W. (2023). Quercetin: A Promising Therapy for Diabetic Encephalopathy through Inhibition of Hippocampal Ferroptosis. Phytomedicine.

[31] Zeng Y.-F., Li J.-Y., Wei X.-Y., Ma S.-Q., Wang Q.-G., Qi Z., Duan Z.-C., Tan L., Tang H. (2023). Preclinical evidence of reno-protective effect of quercetin on acute kidney injury: A meta-analysis of animal studies. Front. Pharmacol..

[32] Li W., Zhang L., Xu Q., Yang W., Zhao J., Ren Y., Yu Z., Ma L. (2022). Taxifolin Alleviates DSS-Induced Ulcerative Colitis by Acting on Gut Microbiome to Produce Butyric Acid. Nutrients.

[33] Selivanova I.A., Tyukavkina N.A., Kolesnik Y.A., Nesterov V.N., Kuleshova L.N., Khutoryanskii V.A., Bazhenov B.N., Saibotalov M.Y. (1999). Study of the crystalline structure of dihydroquercetin. Pharm. Chem. J..

[34] Nifant’eV E.E., Koroteev M.P., Kaziev G.Z., Uminskii A.A., Grachev A.A., Men’sHov V.M., Tsvetkov Y.E., Nifant’eV N.E., Bel’sKii V.K., Stash A.I. (2006). On the problem of identification of the dihydroquercetin flavonoid. Russ. J. Gen. Chem..

[35] Terekhov R.P., Selivanova I.A., Tyukavkina N.A., Shylov G.V., Utenishev A.N., Porozov Y.B. (2019). Taxifolin tubes: Crystal engineering and characteristics. Acta Crystallogr. Sect. B Struct. Sci..

[36] Wu W., Wang L., Li W., Zu Y., Wang L., Zhang Y., Zhao X. (2019). Preparation and Characterization of Taxifolin form II by Antisolvent Recrystallization. Chem. Eng. Technol..

[37] Terekhov R.P., Selivanova I.A., Tyukavkina N.A., Ilyasov I.R., Zhevlakova A.K., Dzuban A.V., Bogdanov A.G., Davidovich G.N., Shylov G.V., Utenishev A.N. (2020). Assembling the Puzzle of Taxifolin Polymorphism. Molecules.

[38] Moura F.C.S., Pinna N., Vivani R., Nunes G.E., Schoubben A., Bresolin T.M.B., Bechold I.H., Ricci M. (2021). Exploring Taxifolin Polymorphs: Insights on Hydrate and Anhydrous Forms. Pharmaceutics.

[39] Kumara K., Jyothi M., Kouser S., Kumar A.U., Warad I., Khanum S.A., Lokanath N.K. (2023). Structural investigations and theoretical insights of a polymethoxy chalcone derivative: Synthesis, crystal structure, 3D energy frameworks and SARS CoV-2 docking studies. J. Mol. Struct..

[40] Xing W., Yu H., Zhang B., Liu M., Zhang L., Wang F., Gong N., Lu Y. (2022). Quantitative Analysis the Weak Non-Covalent Interactions of the Polymorphs of Donepezil. ACS Omega.

[41] Mukherjee A. (2015). Building upon Supramolecular Synthons: Some Aspects of Crystal Engineering. Cryst. Growth Des..

[42] Sheldrick G.M. (2015). SHELXT–Integrated space-group and crystal-structure determination. Acta Crystallogr. Sect. A Found. Adv..

[43] Spackman M.A., Jayatilaka D. (2009). Hirshfeld surface analysis. CrystEngComm.

[44] Spackman P.R., Turner M.J., McKinnon J.J., Wolff S.K., Grimwood D.J., Jayatilaka D., Spackman M.A. (2021). CrystalExplorer: A program for Hirshfeld surface analysis, visualization and quantitative analysis of molecular crystals. J. Appl. Crystallogr..

[45] Macrae C.F., Sovago I., Cottrell S.J., Galek P.T.A., McCabe P., Pidcock E., Platings M., Shields G.P., Stevens J.S., Towler M. (2020). Mercury 4.0: From visualization to analysis, design and prediction. J. Appl. Crystallogr..

[46] Turner M.J., Grabowsky S., Jayatilaka D., Spackman M.A. (2014). Accurate and Efficient Model Energies for Exploring Intermolecular Interactions in Molecular Crystals. J. Phys. Chem. Lett..

[47] Mackenzie C.F., Spackman P.R., Jayatilaka D., Spackman M.A. (2017). CrystalExplorer model energies and energy frameworks: Extension to metal coordination compounds, organic salts, solvates and open-shell systems. IUCrJ.

[48] Yuan P., Wang Y., Wang W., An Q., Li S., Zhang B., Zhou J., Hu K., Zhang L., Yang D. (2023). Characterization, Analysis, and Theoretical Calculation Studies of Solvates and Cocrystals of Betulin: An Exploration of the Boundary between Solvates and Cocrystals. Cryst. Growth Des..

[49] Gong N., Zhang G., Jin G., Du G., Lu Y. (2016). Polymorphs and Versatile Solvates of 7-Hydroxyisoflavone. J. Pharm. Sci..

[50] Jyothi K.L., Kumara K., Hema M.K., Gautam R., Row T.G., Lokanath N.K. (2020). Structural elucidation, theoretical insights and thermal properties of three novel multicomponent molecular forms of gallic acid with hydroxypyridines. J. Mol. Struct..

[51] Mahesha, Hema M.K., Karthik C.S., Pampa K.J., Mallu P., Lokanath N.K. (2021). Solvent induced mononuclear and dinuclear mixed ligand Cu(II) complex: Structural diversity, supramolecular packing polymorphism and molecular docking studies†. New J. Chem..

[52] Zhang T., Yang S., Zhang B., Yang D., Lu Y., Du G. (2022). Insights Into the Properties of Amygdalin Solvatomorphs: X-ray Structures, Intermolecular Interactions, and Transformations. ACS Omega.

[53] Liu M., Xing W., Zou Y., Hu K., Gong N., Lu Y., Du G. (2024). Understanding solvent effects on solvatomorphisms of donepezil-maleic acid. J. Mol. Struct..

[54] Ahmadi S., Mondal P.K., Mirmehrabi M., Rohani S. (2021). Desolvation of dasatinib methanolate: An improved anhydrous polymorph. CrystEngComm.

[55] Yuan L., Horosanskaia E., Engelhardt F., Edelmann F.T., Couvrat N., Sanselme M., Cartigny Y., Coquerel G., Seidel-Morgenstern A., Lorenz H. (2018). Solvate Formation of Bis(demethoxy)curcumin: Crystal Structure Analyses and Stability Investigations. Cryst. Growth Des..

[56] Liu W., Hou B., Huang X., Zong S., Zheng Z., Li S., Zhao B., Liu S., Zhou L., Hao H. (2021). Influence of intermolecular interactions and crystal structure on desolvation mechanisms of solvates. CrystEngComm.

[57] Chęcińska L., Jóźwiak A., Ciechańska M., Paulmann C., Holstein J.J., Dittrich B., Małecka M. (2018). Quantifying intermolecular interactions for isoindole derivatives: Substituent effect vs. crystal packing. Z. Krist. Mater..

[58] Kaspiaruk H., Chęcińska L. (2022). A comparison of three crystalline forms of miconazole: Solvent-free, ethanol monosolvate and hemihydrate. Acta Crystallogr. Sect. C Struct. Chem..

[59] Ge S., Fu M., Cai Z., Gu D., Ma Y., Wang H., Ge M., Wang Y. (2024). Study of the Polymorphic Transformation Mechanism and Crystal Habits Control of Peramivir from Dihydrate to Trihydrate. Cryst. Growth Des..

[60] Demetzos C. (2008). Differential Scanning Calorimetry (DSC): A tool to study the thermal behavior of lipid bilayers and liposomal stability. J. Liposome Res..

[61] Krysa M., Szymańska-Chargot M., Zdunek A. (2022). FT-IR and FT-Raman fingerprints of flavonoids—A review. Food Chem..

[62] Zhang Y., Yu J., Dong X.-D., Ji H.-Y. (2017). Research on Characteristics, Antioxidant and Antitumor Activities of Dihydroquercetin and Its Complexes. Molecules.

[63] Heneczkowski M., Kopacz M., Nowak D., Kuźniar A. (2001). Infrared spectrum analysis of some flavonoids. Acta Pol. Pharm. Drug Res..

