# Supplementary material for: X-Ray Structures, Intermolecular Interactions, and Structural Transformations of Dihydroquercetin Solvates and Polymorphs
Source: Pharmaceutics. 2025 Nov 23;17(12):1512. doi: 10.3390/pharmaceutics17121512 (PMC12736233; doi:10.3390/pharmaceutics17121512)
Supplement: Supplementary file 1 [file pharmaceutics-17-01512-s001.zip › pharmaceutics-3947061-supplementary.pdf]

Supporting information

X-ray Structures, Intermolecular Interactions, and Structural Transformations of Dihydroquercetin Polymorphs

Xin Meng [1], Yao Zou [1], Shiying Yang [1], Cheng Xing [1], Ningbo Gong [1,*], Guanhua Du [2] and Yang Lu [1,*]

1 Beijing Key Laboratory of Polymorphic Drugs, Institute of Materia Medica, Chinese Academy of Medical Sciences and Peking Union Medical College, Beijing 100050, China

2 Beijing City Key Laboratory of Drug Target Identification and Drug Screening, Institute of Materia Medica, Chinese Academy of Medical Sciences and Peking Union Medical College. Beijing 100050, China

* Correspondence: gnb@imm.ac.cn (N. B. Gong); luy@imm.ac.cn (Y. Lu).

Table and Figure of Contents

Table S1: Hydrogen bond geometrical parameters of crystal structures

Table S2: Molecular Pairwise Interaction Energies (kJ/mol) Derived from the Energy Framework Calculation for S1 of DHQ

Table S3: Molecular Pairwise Interaction Energies (kJ/mol) Derived from the Energy Framework Calculation for S2 of DHQ.

Table S4: Molecular Pairwise Interaction Energies (kJ/mol) Derived from the Energy Framework Calculation for S3 of DHQ.

Table S5: Molecular Pairwise Interaction Energies (kJ/mol) Derived from the Energy Framework Calculation for S4 of DHQ.

Table S6: Molecular Pairwise Interaction Energies (kJ/mol) Derived from the Energy Framework Calculation for S5 of DHQ.

Table S7: FT−IR spectral frequencies of DHQ solvates and polymorphs.

Table S1: Hydrogen Bonds in the Crystal Structures.

H bond	d(D−H) / Å	d(H…A) / Å	d(D…A) / Å	Symmetry Code
		**S1**		
O3−H3…O2	0.82	2.40	2.697	
O3−H3…O8	0.82	2.04	2.828	−x,−y+2,−z+1
O4−H4…O2	0.82	1.60	2.639	
O4−H4…O2	0.82	2.43	2.631	−x, −y+1, −z+2
O5−H5…O7	0.82	2.05	2.808	−x+2, −y+1, −z+1
O6−H6…O7	0.82	2.31	2.676	
O6−H6…O3	0.82	1.99	2.738	−x+1, −y+2, −z+1
O7−H7…O8	0.82	1.87	2.661	
O8−H8A…O4	0.85	2.05	2.834	x, y+1, z−1
O8−H8B…O5	0.85	2.40	3.407	−x+1, −y+1, −z+1
		**S2**		
O3 −H3A…O2	0.82	2.38	2.713	
O3 −H3A…O2	0.82	2.02	2.755	−x+2, −y+1, −z+1
O4 −H4…O2	0.82	1.92	2.637	
O5 −H5…O8	0.82	1.89	2.703	
O6 −H6…O7	0.82	2.01	2.829	x+1/2, −y+1/2, z+1/2
O7 −H7…O6	0.82	2.22	2.669	
O7 −H7…O1	0.82	2.34	2.954	x+1/2, −y+1/2, z−1/2
O8 −H8A…O3	0.85	2.02	2.785	x−1, −y, z+1
O8 −H8B…O4	0.85	2.19	2.973	−x,−y+1,−z+2

		**S3**		
O2−H2A…O3	0.82	2.29	2.704	
O2−H2A…O3	0.82	2.02	2.772	−x+1, −y+2, −z+1
O4−H4…O3	0.82	1.90	2.616	
O5−H5…O6	0.82	1.94	2.756	x, −y ,z+1/2
O5−H5…O7	0.82	2.59	2.89	x, −y ,z+1/2
O6−H6…O8	0.82	1.89	2.689	
O7−H7…O5	0.82	2.05	2.809	−x+2/3, −y+1/2, −z+1
O8−H8A…O2	0.85	2.10	2.924	x, −1+y, z
O8−H8B…O9	0.85	2.38	2.77	−x+1,−y,−z+1
O9−H9A…O8	0.85	1.94	2.77	−x+1,−y,−z+1
O9−H9B…O4	0.85	2.00	2.729	
		**S4**		
O3−H3B…O7B	0.82	2.53	2.992	−x+1, −y+2, −z+1
O3−H3B…O6	0.82	1.68	2.774	−x+1, −y+2, −z+1
O4−H4…O2	0.82	1.92	2.642	
O4−H4…O5	0.82	2.51	2.864	−x+2, y+1/2, −z+1/2
O5−H5 …O9	0.82	1.94	2.761	
O6−H6A …O8	0.82	2.57	2.845	x, y, z+1
O7B −H7B…O8	0.82	2.13	2.464	
O7A −H7A…O9	0.82	2.40	2.987	−x+1, y+1/2, −z+1/2
O9 −H9A…O8	0.85	2.44	2.874	−x+1, y−1/2, −z+1/2
O9 −H9A…O3	0.85	2.12	2.918	x, −y+2/3, z+1/2
O9 −H9B…O10	0.85	1.83	2.681	
O10 −H10A…O2	0.85	2.40	3.057	x, y−1, z
O10 −H10A…O3	0.85	2.24	2.984	x, y−1, z
O10 −H10B…O4	0.85	2.00	2.825	−x+2, −y+1, −z+1
		**S5**		
O3−H3A…N1	0.82	2.02	2.767	
O4−H4…O1	0.82	2.56	2.998	x, −y+1/2, z−1/2
O4−H4…O2	0.82	1.90	2.617	
O5−H5…O2	0.82	2.11	2.785	x+1, −y+1/2, z+1/2
O5−H5…O3	0.82	2.47	3.208	x+1, −y+1/2, z+1/2
O6−H6…O4	0.82	1.95	2.762	x−1, −y+1/2, z+1/2
O7−H7…O6	0.82	2.26	2.701	
O7−H7…O6	0.82	2.16	2.867	−x, −y+1, −z+2

Table S2: Molecular Pairwise Interaction Energies (kJ/mol) Derived from the Energy Framework Calculation for S1 of DHQ.

	N	Symop	R	E_ele	E_pol	E_dis	E_rep	E_tot
	1	x,1+y,z	8.05	-38.2	-6.6	-3.2	30.3	-30.6
	1	x,y,1+z	7.29	-14	-2.1	-3.4	16.9	-10.4
	1	1-x,2-y,1-z	6.86	-3.6	-0.9	-3.4	4.8	-5.3
	1	2-x,1-y,1-z	5.37	-15.9	-2.7	-3.7	15.8	-13.1
	1	1+x,y,1+z	7.45	-1.9	-1	-3.3	2.3	-4.7
	1	-x,1-y,1-z	6.29	-26.2	-8.2	-22.3	39.1	-34.8
	1	1-x,-y,1-z	8.34	-48.6	-7.6	-34.2	64.9	-55.4
	1	2-x,1-y,-z	10.13	-7.7	-1.4	-7.8	10.1	-11.4
	1	-x,2-y,-z	13.35	-4.7	-0.6	-6	8.1	-7.2
	1	1+x,1+y,z	8.38	-1	-0.3	-1.7	0.4	-2.8
	1	1-x,1-y,1-z	5.52	-13.1	-1.5	-31.7	28.8	-33.2
	2	-1+x,y,z	5.38	-4.7	-2.6	-32.5	17.3	-30.8
	1	-x,-y,1-z	9.96	6.6	-1.3	-6.7	14.2	6.5
	1	1-x,1-y,1-z	4.91	-2.1	-0.5	-1.9	0.4	-4.3
	1	-x,2-y,1-z	8.92	-5.4	-0.2	-0.7	0.1	-6.3
	1	2-x,-y,1-z	9.88	1.4	-0.5	-3.8	0.1	-2.7
	1	1-x,1-y,-z	8.46	-7.6	-1.5	-24.8	18.4	-24.2
	2	x,-1+y,1+z	14.6	-2.4	-0.2	-1.7	0.1	-4.2

Table S3: Molecular Pairwise Interaction Energies (kJ/mol) Derived from the Energy Framework Calculation for S2 of DHQ.

	N	Symop	R	E_ele	E_pol	E_dis	E_rep	E_tot
	1	-1+x,y,1+z	7.92	-43.3	-8.1	-4	35	-35.4
	1	-	4.52	-21.2	-3.4	-6.4	23.7	-17.7
	1	1-x,1-y,1-z	6.8	-13.3	-1.7	-3.7	10.8	-12.6
	1	-1+x,y,z	5.33	0.6	-0.4	-2.1	1.1	-1.4
	1	-x,1-y,1-z	8.35	-33.4	-4.5	-9.8	33	-29.7
	2	1/2+x,1/2-y,1/2+z	8.55	-25.6	-4.7	-9.4	20.7	-27.9
	1	x,y,1+z	6.72	0.9	-0.5	-3.1	1.8	-1.7
	2	-1/2+x,1/2-y,1/2+z	8.83	-7.3	-1.2	-14.5	12.3	-16.5
	1	1-x,1-y,-z	12.35	-6	-1.1	-6.5	6.7	-10.2
	1	-x,1-y,1-z	6.88	-1.3	-0.5	-2.2	0.9	-3.3
	2	-1+x,y,z	4.83	-14.3	-2.6	-48.1	35.5	-47.8
	2	-1+x,y,1+z	11.78	-2.8	-0.7	-5.5	3.1	-7.1
	2	x,y,-1+z	10.29	0.4	-0.4	-8.3	3.8	-6.6
	1	1-x,1-y,1-z	7.3	-2.9	-1	-15.7	2.5	-18
	2	3/2+x,1/2-y,-1/2+z	11.38	-1.6	-0.2	-2.5	0.2	-4.1
	1	2-x,1-y,-z	13.87	-3.3	-0.3	-1.7	0	-5.3
	1	1+x,y,z	7.7	-0.2	-0.1	-0.2	0	-0.5
	1	-1+x,y,1+z	9.59	0.2	0	-0.1	0	0

Table S4: Molecular Pairwise Interaction Energies (kJ/mol) Derived from the Energy Framework Calculation for S3 of DHQ.

	N	Symop	R	E_ele	E_pol	E_dis	E_rep	E_tot
	1	-	4.7	-14.5	-2.8	-5.8	15.5	-14.5
	1	x,-1+y,z	4.86	-34.4	-6.4	-4.6	32.3	-27.2
	1	-	7.43	-57.4	-3.7	-9.4	129	-0.9
	1	1-x,-y,1-z	7.18	-1.3	-0.3	-2.1	1	-3.1
	2	x,2-y,-1/2+z	12.55	-28.5	-6.1	-7.2	26.9	-26.2
	1	1-x,-y,1-z	8.57	-29.4	-3.8	-10	33	-25.3
	1	3/2-x,1/2-y,1-z	6.37	-38.2	-5.4	-23.8	50.9	-40.9
	2	3/2-x,-1/2+y,5/2-z	14.06	-7.9	-0.8	-3.1	0.9	-11
	1	1-x,1-y,1-z	7.02	-0.9	-0.6	-2.2	0.5	-3.4
	1	x,1-y,1/2+z	8.83	-6.6	-0.5	-1.2	0.3	-8.1
	1	1-x,1-y,1-z	6.12	0.4	-0.4	-5.8	4.2	-3.2
	2	x,-1+y,z	4.81	-16.2	-2.7	-46.1	30.5	-50
	1	1-x,1-y,1-z	7.04	-6	-1	-16.9	4.4	-21.6
	1	1-x,2-y,1-z	7.51	-0.7	-0.2	-1.5	0.3	-2.1
	1	1-x,-1+y,1/2-z	8.48	0	-0.1	-0.3	0	-0.3
	1	x,-1+y,z	9.03	0.4	-0.1	-1.4	0.2	-1
	1	3/2-x,-1/2-y,1-z	6.43	-5.7	-0.9	-19.2	6.4	-23
	1	x,1+y,z	8.66	0.4	0	-1.1	0.1	-0.7
	1	x,-y,1/2+z	8.96	0.4	-0.1	-0.4	0	-0.1
	2	x,1-y,-1/2+z	11.63	-3.1	-1.5	-10	5	-11.7
	1	1-x,-y,1-z	8.05	-0.4	0	-0.7	0	-1.1

Table S5: Molecular Pairwise Interaction Energies (kJ/mol) Derived from the Energy Framework Calculation for S4 of DHQ.

	N	Symop	R	E_ele	E_pol	E_dis	E_rep	E_tot
	1	x,-1/2-y,-1/2+z	5.1	-13.8	-2.4	-4	12.1	-13.6
	1	-	6.19	-36.7	-6.3	-3.8	24.7	-32.5
	1	-x,1-y,1-z	7.27	-17.4	-2.4	-3.4	19.5	-12.7
	1	x,1+y,z	5.28	-24.5	-3.3	-4.3	12.9	-24.5
	1	1-x,1/2+y,1/2-z	7.71	-6.4	-1.8	-3.9	12.9	-4.5
	2	x,y,-1+z	7.12	7	-1.7	-5.9	1.8	0.5
	1	1-x,-y,1-z	8.41	-16.4	-3.7	-29.7	42.2	-27.3
	2	-x,1/2+y,1/2-z	11.79	-5.1	-1	-5.9	6.4	-8.3
	1	1-x,1-y,1-z	8.95	-5.3	-0.4	-1.4	0.7	-6.7
	1	1-x,-y,2-z	8.94	6.6	-2.7	-14.4	7.8	-5.6
	1	-x,1/2+y,1/2-z	7.85	1.7	-0.3	-1.9	0.4	-0.2
	1	-x,1-y,1-z	12.87	-1	-0.4	-5.6	3	-5.3
	1	x,1/2-y,-1/2+z	7.35	-3.8	-0.2	-1.2	0.1	-5.1
	1	-x,1-y,1-z	8.74	-2	-0.1	-1	0.1	-3.1
	1	1-x,1-y,1-z	8.09	2.3	-0.2	-0.9	0	1.2
	2	x,3/2-y,1/2+z	3.96	-20.6	-2.8	-58.8	47.4	-59.6
	1	1-x,1/2+y,1/2-z	9.03	-1.5	-0.2	-0.6	0	-2.2

Table S6: Molecular Pairwise Interaction Energies (kJ/mol) Derived from the Energy Framework Calculation for S5 of DHQ.

	N	Symop	R	E_ele	E_pol	E_dis	E_rep	E_tot
	1	-x,-1/2+y,3/2-z	9.06	-5.3	-1	-3.1	10.1	-3.9
	1	-	6.07	-26.6	-5.9	-15.4	45.3	-23.5
	1	1+x,y,z	5.99	-3.5	-1.7	-7.2	7.8	-8.5
	1	2-x,1-y,-z	12.99	-18.5	-1.8	-6.9	18.5	-17
	2	-1+x,1/2-y,-1/2+z	8.84	-32.6	-6.7	-8.8	18	-37.2
	2	-1+x,1/2-y,1/2+z	8.96	-28.4	-5.6	-8.5	24.1	-28.7
	2	x,1/2-y,1/2+z	7.26	-2.9	-1.7	-16	9.6	-15.3
	1	1-x,-1/2+y,3/2-z	7.09	-1.5	-0.8	-5.2	2.4	-6.3
	2	-x,-1/2+y,3/2-z	12.42	-0.9	-0.1	-3.8	0.8	-4.5
	1	1-x,1-y,1-z	5.61	-4.7	-1	-4.8	0.7	-9.8
	1	2-x,1-y,1-z	7.18	-2.4	-0.3	-1.5	0.1	-4
	1	1+x,1/2-y,1/2+z	10.25	-2.6	-0.4	-0.9	0.1	-3.9
	1	2-x,1-y,-z	9.93	1.9	-0.1	-0.4	0	1.4
	1	1-x,1-y,-z	10.91	-4.4	-0.5	-13.9	9.9	-13.6
	2	-1+x,y,z	5.15	-3.2	-2.8	-40.3	22.1	-35.6
	2	1-x,1/2+y,3/2-z	12.24	-1	-0.2	-3.1	0.3	-4.1
	2	-2+x,1/2-y,1/2+z	12.68	-0.8	-0.1	-0.4	0	-1.3
	1	2+x,1/2-y,1/2+z	12.49	-0.9	0	-0.1	0	-1.1

Table S7: FT−IR spectral frequencies of DHQ solvates and polymorphs.

**Solvate**	**–O–H Stretch**(cm^{-1})	**–C=O Strech**(cm^{-1})
S1	3225	1603
S2	3412, 3078	1654
S3	3360, 2988	1657
S5	3364	1606
S6	3313	1626
